# Immunosuppressive therapy and COVID‐19 infection in patients with NMOSD

**DOI:** 10.1002/iid3.1128

**Published:** 2024-01-16

**Authors:** Un Wai Choi, Xiwen Ai, Hongyan Li, Yong Hao, Xiaoying Yao, Yangtai Guan

**Affiliations:** ^1^ Department of Neurology, Ren Ji Hospital Shanghai Jiao Tong University School of Medicine Shanghai China

**Keywords:** COVID‐19, human umbilical cord mesenchymal stem cell (hUC‐MSC), immunosuppressant, neuromyelitis optica spectrum disorder (NMOSD)

## Abstract

**Introduction:**

To evaluate whether treated with immunosuppressants in neuromyelitis optica spectrum disorder (NMOSD) shows an effect on the severity and outcomes of COVID‐19 Omicron variant.

**Methods:**

This is a substudy of a single‐center clinical trial involving human umbilical cord mesenchymal stem cells (hUC‐MSCs) in NMOSD patients. NMOSD patients with hUC‐MSCs treatment, NMOSD patients without hUC‐MSCs treatment, and matched healthy controls (HC) were included. Demographic information, NMOSD‐related clinical features, comorbidities, use of disease‐modifying therapy, COVID‐19 vaccination status, COVID‐19 clinical features, COVID‐19 clinical outcomes, and NMOSD‐related disease activity were obtained through online questionnaires or phone calls.

**Results:**

The majority of NMOSD patients received long‐term treatment with mycophenolate mofetil (68.8%) or azathioprine (22.9%), and 50% received oral glucocorticoid. During the epidemic, 97.4% of NMOSD patients infected with COVID‐19 had asymptomatic or mild forms, with only two patients (2.6%) requiring hospitalization. None of these patients required tracheal intubation or admission to the intensive care unit. Clinical symptoms were found to be more prevalent in HC groups. Additionally, the HC groups had higher fever‐recorded temperatures. NMOSD patients who received hUC‐MSCs treatment had shorter disease duration than patients who did not receive hUC‐MSCs treatment.

**Discussion:**

Immunosuppressant‐treated patients with NMOSD have a similar risk of COVID‐19 infection as the general population, but the disease duration is shorter and the clinical symptoms are less severe. Among our NMOSD patients who received hUC‐MSCs treatment, COVID‐19 outcomes were favorable, with no increased risk of severe COVID‐19. Prospective studies on immunotherapies are needed to help determine best treatment practices.

## Graphical abstract

Immunosuppressant‐treated neuromyelitis optica spectrum disorder (NMOSD) patients have a similar risk of COVID‐19 infection to the general population. In immunosuppressant‐treated NMOSD patients, COVID‐19 disease duration is shorter and clinical symptoms are less severe. In addition, NMOSD patients treated with hUC‐MSC had favorable COVID‐19 outcomes with no increased risk of severe COVID‐19.

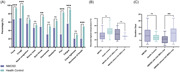

## 1 INTRODUCTION

Coronavirus disease 2019 (COVID‐19) had been declared a global pandemic from March 2020 to May 2023. It is highly contagious among humans and, in moderate to severe cases, causes bilateral interstitial pneumonia with associated respiratory failure.^1^ COVID‐19 had compelled governments all over the world to implement social distancing or lockdown measures. China's strict zero‐COVID policy kept infections at bay, since the Chinese CDC announced that the policy ended in December 2022, the coronavirus Omicron variant has spread widely throughout the country.

Neuromyelitis optica spectrum disorder (NMOSD) is a severe relapsing and disabling central nervous system autoimmune disease. Its optimal first‐line treatment strategy remains unclear. Immunosuppression is frequently used to improve the prognosis of NMOSD.^2^, ^3^ In the early days of the pandemic, little was known about the outcome of COVID‐19 in patients with NMOSD, particularly when immunosuppressive therapy was used.

Immunosuppression increased the incidence and severity of many infectious diseases. According to a previous study, rituximab increased the risk of severe COVID‐19 in rheumatoid arthritis.^4^ Nevertheless, current recommended guidelines worldwide suggest that immunosuppression treatment be continued in patients who require it, with the exception of possibly high‐dose corticosteroid therapy and patients with associated risk factors for severe COVID‐19 disease.^5^ However, the exact effect of immunosuppressives on the severity of COVID‐19 is unknown. Human umbilical cord‐mesenchymal stem cells (hUC‐MSCs) can regulate the immune response, promote tissue repair, and increase regeneration.^6^ The current reviews demonstrated that hUC‐MSCs therapy can improve pulmonary function and imaging appearance, as well as reduce COVID‐19 mortality in critical COVID‐19 patients.^7^ Studies on the efficacy of hUC‐MSC in treating different types of diseases reported preliminary results.^8^, ^9^, ^10^ No toxicities were reported in these studies. hUC‐MSCs are a promising candidate for cell‐based therapy. We conduct a prospective study for an open‐labeled, prospective, multicenter, randomized, placebo‐controlled clinical trial on safety and effectiveness of hUC‐MSCs in treating NMOSD. This is a substudy of the trial. NMOSD patients with hUC‐MSCs treatment have received three to four times of hUC‐MSCs intravenous infusions every 3 months besides the primary immunosuppressive drugs from June 2020 to December 2022. The dosages are 1, 2, or 5 × 10^6^ MSCs per kg of body weight.

The objective of this study was to evaluate the risk and clinical outcome of COVID‐19 among NMOSD patients with immunosuppressant therapy and hUC‐MSCs treatment. We aim to demonstrate the clinical safety and efficacy of hUC‐MSC in treating NMOSD. Despite the insights from early studies, the interplay between immunosuppression and virus infection in patients with NMOSD is a pressing clinical question.

## 2 METHOD

This is an observational case–control substudy of a single‐center, prospective cohort study on the use of human umbilical cord mesenchymal stem cells (hUC‐MSCs) in the treatment of NMOSD.^11^ A survey was used to evaluate patients who were recruited in hUC‐MSCs trials and those who did not, as well as a healthy control group, from December 7, 2022 to January 14, 2023. Demographics, NMOSD‐related clinical features, comorbidities, and use of disease‐modifying therapy (DMT), method for the diagnosis of COVID‐19, clinical features of COVID‐19, clinical outcomes of COVID‐19, and NMOSD‐related disease activity were obtained via online questionnaires or phone calls in January 2023. The following were the inclusion criteria: (1) Adult patients (aged 18 years) with NMOSD diagnosed using the 2015 International Panel Criteria (2) confirmed COVID‐19 infection (reverse‐transcription polymerase chain reaction or antigen rapid test) or clinical suspicion of COVID‐19. Patients who did not complete questionnaires were not included in this study. As a health control, we recruit adult participants separately. The questionnaire consisted of the following main categories: (1) demographic and clinical data (age, sex, weight, height, comorbidities, and current immunotherapy), (2) COVID‐19 infection (COVID‐19 laboratory test, disease duration, symptoms), (3) effects of the pandemic (changes of immunotherapy), and (4) COVID‐19 vaccination status.

Descriptive statistics were used to describe the demographic and basic clinical data. The chi‐square test for categorical variables and the Student *t* test for comparing means in parametric variables were both used to assess the statistical significance of differences between groups. *p* < 0.05 were considered significant. IBM SPSS Statistics (Version 26) was used for all analyses.

## 3 RESULTS

Demographic and clinical characteristics of participants are summarized in Table 1. The vast majority of patients with NMOSD received long‐term treatment of mycophenolate mofetil (68.8%) or azathioprine (22.9%), and 50% of patients received oral glucocorticoid. NMOSD group was further classified as with (*n* = 31) and without (*n* = 25) hUC‐MSCs treatment.

**Table 1:** Demographic and clinical characteristics of the study participants.

	Total (*n* = 91)	Health control (*n* = 35)	NMOSD (*n* = 56)	*p* Value	NMOSD with hUC‐MSCs treatment (*n* = 31)	NMOSD without hUC‐MSCs treatment (*n* = 25)	*p* Value
Age (means ± SD)	43.82 ± 11.19	42 ± 8.48	44.96 ± 12.53	0.221	43.1 ± 12.161	47.28 ± 12.847	0.217
Sex (*n*, %)				0.475			0.711
Female	76 (83.5)	28 (80)	48 (85.7)		26 (83.9)	22 (88)	
Male	15 (16.5)	7 (20)	8 (14.3)		5 (16.1)	3 (12)	
BMI (*n*, %)				0.048			0.047
<18.5	4 (4.4)	1 (2.9)	3 (5.4)		3 (9.7)	0 (0)	
18.5 ≤ BMI < 24	63 (69.2)	30 (85.7)	33 (58.9)		20 (64.5)	13 (52)	
24 ≤ BMI < 28	20 (22)	4 (11.4)	16 (28.6)		8 (25.8)	8 (32)	
≥28	4 (4.4)	0 (0)	4 (7.1)		0 (0)	4 (16)	
Vaccinated (*n*, %)	40 (44)	31 (88.6)	9 (16.1)	<0.001	3 (9.7)	6 (24)	0.000
Vaccinated dose (*n*, %)				<0.001			0.000
1	2 (2.2)	1 (2.9)	1 (1.8)		1 (3.2)	0 (0)	
2	6 (6.6)	3 (8.6)	3 (5.4)		1 (3.2)	2 (8)	
≥3	32 (35.2)	27 (77.1)	5 (8.9)		1 (3.2)	4 (16)	
Developed COVID‐19 clinical symptoms (*n*, %)	78 (85.7)	30 (85.7)	48 (85.7)	1.000	30 (96.8)	18 (72)	0.031
COVID‐19 PCR test	9 (11.5)	1 (3.3)	8 (16.7)		6 (20)	2 (11.1)	
COVID‐19 rapid antigen test	60 (76.9)	29 (96.7)	31 (64.6)		15 (50)	16 (88.9)	
Clinical	9 (11.5)	0 (0)	9 (18.8)		9 (30)	0 (0)	
Smoking (*n*, %)	7 (7.8)	0 (0)	7 (12.5)	0.029	7 (22.6)	2 (8)	0.361
Alcohol use (*n*, %)	2 (2.2)	0 (0)	2 (3.6)	0.258	2 (6.4)	0 (0)	0.196
Comorbidity (*n*, %)							
Hypertension	6 (6.6)	0 (0)	6 (10.7)	0.045	2 (6.4)	4 (16)	0.251
Diabetes	5 (5.5)	0 (0)	5 (8.9)	0.069	2 (6.4)	3 (12)	0.469
Systemic lupus erythematosus	4 (4.4)	0 (0)	4 (7.1)	0.106	1 (3.2)	3 (12)	0.205
Sjogren's syndrome	6 (6.6)	0 (0)	6 (10.7)	0.045	2 (6.4)	4 (16)	0.251
Rheumatoid arthritis	2 (2.2)	0 (0)	2 (3.6)	0.258	0 (0)	2 (8)	0.109
Hypothyroidism	4 (4.4)	0 (0)	4 (7.1)	0.106	4 (9.7)	1 (4)	0.412
Hyperthyroidism	2 (2.2)	0 (0)	2 (3.6)	0.258	1 (3.2)	1 (4)	0.877
Malignancy	2 (2.2)	0 (0)	2 (3.6)	0.427	0 (0)	2 (8)	0.109
Chronic bronchitis	2 (2.2)	0 (0)	2 (3.6)	0.258	0 (0)	2 (8)	0.109
Asthma	1 (1.1)	0 (0)	1 (1.8)	0.427	0 (0)	1 (4)	0.261
Hepatic dysfunction	1 (1.1)	0 (0)	1 (1.8)	0.427	0 (0)	1 (4)	0.261
Autoimmune hepatitis	1 (1.1)	0 (0)	1 (1.8)	0.427	1 (3.2)	0 (0)	0.365
Anemia	1 (1.1)	0 (0)	1 (1.8)	0.427	1 (3.2)	0 (0)	0.365
Hypotension	1 (1.1)	0 (0)	1 (1.8)	0.427	1 (3.2)	0 (0)	0.365
Osteoporosis	1 (1.1)	0 (0)	1 (1.8)	0.427	1 (3.2)	0 (0)	0.365
Hyperlipidemia	1 (1.1)	0 (0)	2 (3.6)	0.258	0 (0)	1 (4)	0.877
Anxiety	1 (1.1)	0 (0)	1 (1.8)	0.427	1 (3.2)	0 (0)	0.365
Other							
Autoimmune disease	0 (0)	0 (0)	0 (0)	‐	0 (0)	0 (0)	‐
Thyroid disease	1 (1.1)	0 (0)	1 (1.8)	0.427	1 (3.2)	0 (0)	0.365
Cardiac disease	0 (0)	0 (0)	0 (0)	‐	0 (0)	0 (0)	‐
Pulmonary disease	0 (0)	0 (0)	0 (0)	‐	0 (0)	0 (0)	‐
Hepatic disease	0 (0)	0 (0)	0 (0)	‐	0 (0)	0 (0)	‐
Renal disease	0 (0)	0 (0)	0 (0)	‐	0 (0)	0 (0)	‐
Neurological disease	0 (0)	0 (0)	0 (0)	‐	0 (0)	0 (0)	‐
NMOSD patient's immunosuppressive treatment during pandemic (*n*, %)							
MMF	‐	‐	33 (68.8)		22 (73.3)	11 (61.1)	0.376
AZA	‐	‐	11 (22.9)		6 (20)	5 (27.8)	0.535
PRED	‐	‐	24 (50)		15 (50)	9 (50)	1
Outcomes of confirmed and probable COVID‐19 in participants							
Hospitalized (*n*, %)	‐	0 (0)	2 (4.2)	0.257	1 (3.3)	1 (5.6)	0.709
NMOSD neurologic manifestations		‐	3 (6.3)	‐	3 (10)	0 (0)	0.158
Relapse			2 (4.2)	‐	2 (6.5)	0 (0)	
Pseudoexacerbation			1 (2.1)	‐	1 (3.2)	0 (0)	

Abbreviations: AZA, Azathioprine; BMI, body mass index; COVID‐19, coronavirus disease 2019; MMF, mycophenolate mofetil; NMOSD, neuromyelitis optica spectrum disorders; PCR, polymerase chain reaction; PRED, prednisolone.

85.7% of participants reported clinical symptoms related to COVID‐19 during epidemic, with no significant difference between NMOSD and HC (*p* = 1.000). There was a significant between subgroups analysis (*p* = 0.031), as 96.8% (*n* = 30) of patients with hUC‐MSCs treatment developed COVID‐19 clinical symptoms, and only 72% (*n* = 18) of patients without hUC‐MSCs treatment developed. The majority of patients (97.4%) had mild COVID‐19 forms that could be managed at home, while two patients (2.6%) required hospitalization. None of these patients required tracheal intubation or intensive care for COVID‐19. Only one patient in the NMOSD group received Azvudine, an antiviral drug, to treat COVID‐19; the remaining patients received symptomatic treatment. Three of 31 patients (10%) treated with NMOSD hUC‐MSCs treatment experienced neurologic manifestations (two relapses and one pseudoexacerbation) during or after COVID‐19 infection, with no significant difference found between groups.

88.4% of participants used a COVID‐19 polymerase chain reaction test (HC: *n* = 1, NMOSD: *n* = 8) or COVID‐19 rapid antigen test (HC: *n* = 29, NMOSD: *n* = 31) as diagnostic test. Clinical diagnostic COVID‐19 was reported by 11.5% of participants, all of whom were in the NMOSD with hUC‐MSCs treatment group (*n* = 9). The overall vaccination rate against COVID‐19 was 44%, of which 88.6% was HC while 16.1% of patients with NMOSD got vaccinated. Thirty‐two participants get full dose vaccination (≥3 doses). The vaccination status has a significant difference between NMOSD and HC (*p* < 0.001).

**Figure 1:**
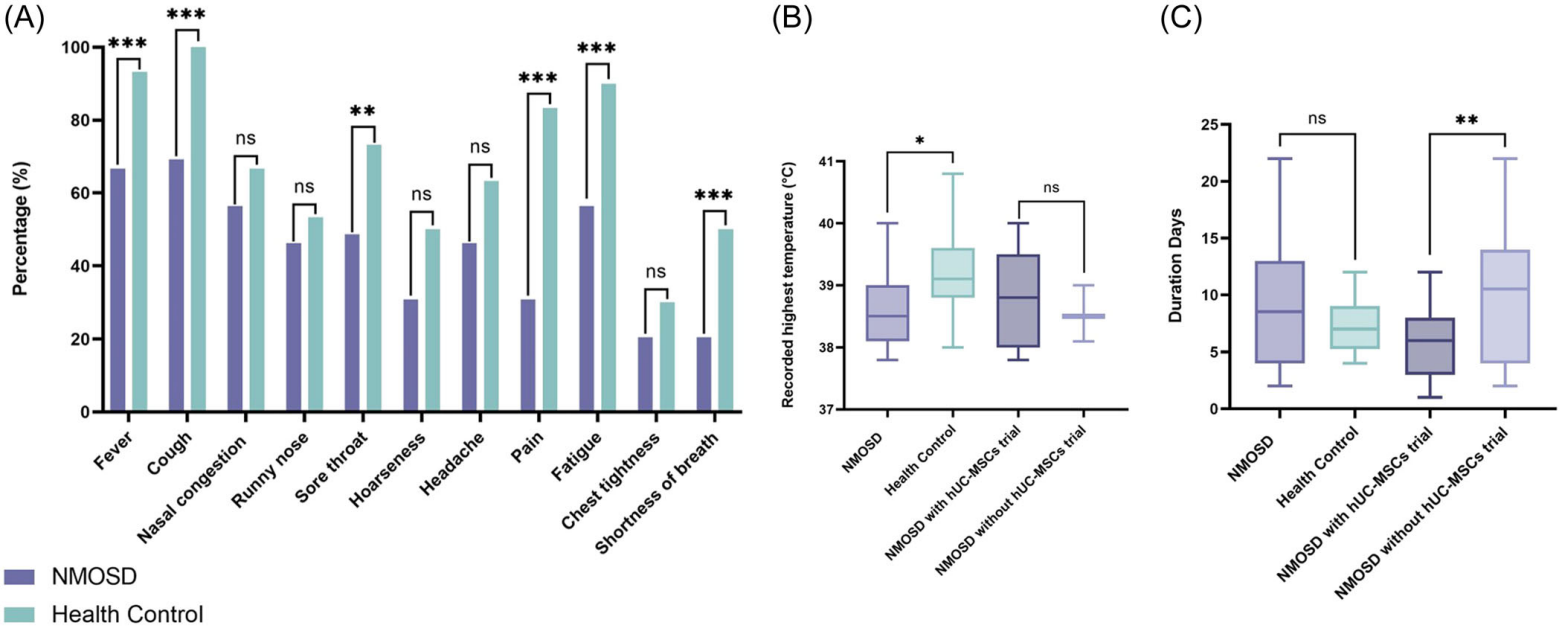
(A) COVID‐19 clinical symptoms comparison between neuromyelitis optica spectrum disorder (NMOSD) and health control group. (B) Comparison of recorded highest temperature between NMOSD and health control group, NMOSD with human umbilical cord mesenchymal stem cells (hUC‐MSCs) trial and NMOSD without hUC‐MSCs trial. (C) Comparison of COVID‐19 disease duration days between NMOSD and health control group, NMOSD with hUC‐MSCs trial and NMOSD without hUC‐MSCs trial.

All of the cases occurred during the omicron wave. General COVID‐19 characteristics in participants are shown in Figure 1. Differences were observed in fever (93.3% vs. 60.4%, *p* = 0.001), cough (100% vs. 66.7%, *p* < 0.001), sore throat (73.3% vs. 43.8%, *p* = 0.011), hoarseness (50% vs. 27.2%, *p* = 0.040), pain (83.3% vs. 31.3%, *p* < 0.001), fatigue (90% vs. 56.3%, *p* = 0.002), and shortness of breath (50% vs. 16.7%, *p* = 0.002) comparing NMOSD and HC, clinical symptoms were found to be more prevalent in HC groups. Furthermore, the highest fever recorded temperatures differed between the NMOSD and HC groups (NMOSD: 38.7 ± 0.7°C vs. HC: 39.2 ± 0.6°C; *p* = 0.011). The number of days the disease persisted did not differ between NMOSD and HC (HC: 8.22 ± 0.99; NMOSD: 7.55 ± 4.86; *p* = 0.513), though NMOSD with hUC‐MSCs treatment was significantly shortened (5.88 ± 3.14) than NMOSD without hUC‐MSCs treatment (9.94 ± 5.92) (*p* = 0.0051).

## 4 DISCUSSION

In this study, the incidences of COVID‐19 was found to be comparable in individuals with NMOSD and HCs. The clinical characteristics of COVID‐19 were found to be discrepancies in NMOSD with hUC‐MSCs treatment, in group without hUC‐MSCs treatment, and in HCs. The prevalence of COVID‐19 in NMOSD patients was found to be 1.2% till July 2021.^12^ A Singaporean study found COVID‐19 Omicron BA.1/2 wave infection in vaccinated patients with MS, NMOSD, and MOGAD was 23.4% over a 4‐month period.^13^ 85.7% of NMOSD patients reported that had developed COVID‐19 clinical symptoms in our study. Despite the relatively high prevalence rate in our cohort, there is no difference between NMOSD group and HC group. The NMOSD group had a significantly lower incidence of COVID‐19‐related clinical symptoms (cough, fever, sore throat, hoarseness, pain, fatigue, and shortness of breath) than the HC group. Fever peak temperature was significantly lower in the NMOSD group than in the HC group (38.7°C vs. 39.2°C). The mean COVID‐19 disease duration was not different between the NMOSD and HC groups (7.55 vs. 8.22 days). Although patients receiving hUC‐MSCs experienced COVID‐19 clinical symptoms in 96.8% of cases, the disease duration was significantly shorter in the hUC‐MSCs trial NMOSD group than was in the non‐hUC‐MSCs trial NMOSD group (5.88 vs. 9.94 days). This could be related to the effectiveness of hUC‐MSCs in treating COVID‐19's hyperactive immune response.^14^ hUC‐MSCs therapy can suppress excessive immune system activation and promote endogenous repair through enhancements to the microenvironment.^15^ Study indicated that recipients of hUC‐MSCs had higher levels of soluble TNFR2 suggesting that binding of soluble TNFR2 to tumor necrosis factor (TNF) would inhibit TNF cytotoxicity and reduce inflammation in COVID‐19.^16^

In comparison to another study in which 89% of NMOSD patients received COVID‐19 vaccination, the vaccination rate in our study (16.1%) was relatively low.^17^ This may be because the voluntary COVID‐19 vaccination in China was initially registered through the workplace, and only healthy individuals without contraindications were encouraged to register. It can be said that the willingness of the NMOSD population to be vaccinated would be low before the strict zero‐COVID policy in China ended.

Studies revealed that individuals with comorbidity had a significantly greater prevalence of COVID‐19 hospitalization than the general population. More than 75% of participants in the ISARIC4C study, which enrolled COVID‐19 hospitalized patients in the United Kingdom, had at least one comorbidity.^18^ Patients with cardiac disease, pulmonary disease, chronic kidney disease, chronic neurological disorders, dementia, obesity, cancer, liver disease had an elevated risk of in‐hospital mortality.^19^ Although organ transplant recipients are one of the populations most at risk of COVID‐19 death, a study of a large US COVID‐19 database indicated that immunosuppressive therapy before hospitalization (for any cause, including transplant) was not related to COVID‐19 in‐hospital mortality.^20^

Long‐term immunosuppressants and/or steroids are used to manage NMOSD in the chronic phase.^3^ The effect of immunosuppressive therapies on COVID‐19 is particularly concerning. The results of this study suggest that treatment with DMT or steroids has been linked to reducing COVID‐19 symptoms and shortening the duration of the disease without increasing the risk of severe and critical illness and hospitalization in NMOSD patients. A Chinese cohort of NMOSD patients, with 69.6% of patients using DMT show no increased risk of COVID‐19 regardless of therapeutic regimen.^21^ An Australian study also found that immunosuppressed patients were not at significantly increased risk of COVID‐19 infection.^22^ Furthermore, an overexuberant host response characterized by elevated interleukin‐6 (IL‐6) and other proinflammatory cytokines was associated with poor outcomes in severe COVID‐19 cases.^23^

Immunosuppressive therapy has been used in the treatment of COVID‐19 patients with hyperinflammation and cytokine storm syndrome (CSS). The inhibition of the JAKs pathway, TNF, IL‐1, and granulocyte‐macrophage colony‐stimulating factor was used to control COVID‐19‐related CSS. Immunosuppressive therapy has shown promising results in the treatment of CSS.^24^ Additionally, Azathioprine was associated with a reduced risk of invasive ventilation in COVID‐19 patients. Yet, treatment with rituximab appears to increase patients' risk of severe COVID‐19.^20^ Anti‐CD20 therapy (rituximab, ocrelizumab, and ofatumumab) was linked with higher COVID‐19 experience and severity.^25^, ^26^ Anti‐CD19 antibody monotherapy (Inebilizumab) also produced similar outcomes to COVID‐19, since antibody generation and responses to COVID‐19 vaccinations were hampered when treated with B‐cell‐depleting therapies.^27^ Anti‐CD20 and anti‐CD19 therapy may increase the incidence of COVID‐19 in NMOSD patients, but this is questionable and requires further research.

This is an observational case–control substudy of an experimental single‐center, prospective clinical trial on the use of hUC‐MSCs in the treatment of NMOSD. We are aware that the study has limitations. First, this study was conducted at a single center with small sample size, which may affect the generalizability of the results. With a small sample size, it is difficult to make accurate predictions or to draw any conclusive conclusions that can be applied to a larger population. Furthermore, the study focuses on a single center limiting its external validity. The findings may not be representative of other regions or populations because healthcare practices, lifestyles, and environmental factors vary. Although selection criteria were clearly defined in the clinical trial protocol to ensure that selection bias was controlled, the use of an online questionnaire or telephone call to collect data could lead to self‐selection bias and recall bias, as participants may not accurately remember or report symptoms or disease activity. Future research is needed to address these limitations and potential biases.

In conclusion, immunosuppressive treatment for NMOSD patients has a similar risk of COVID‐19 infection as the general population, but the disease duration is shorter and the clinical symptoms are less severe. Though our findings indicate that long‐term immunosuppressive therapy appears to be safe, prospective studies on immunotherapies that control for a variety of variables, including acute immunotherapy regimen and disease refractoriness, are needed to help determine best treatment practices.

## LIMITATION

Limitation of this study is the small sample size, which may affect the generalizability of the results. With a small sample size, it is difficult to make accurate predictions or draw conclusive conclusions that can be applied to a larger population. Furthermore, the study's regional focus on Shanghai limits its external validity. The findings may not be representative of other regions or populations because healthcare practices, lifestyles, and environmental factors vary greatly. As a result, more research into potential biases or variables is required.

## AUTHOR CONTRIBUTIONS

**Un Wai Choi**: Formal analysis; visualization; writing—original draft. **Xiwen Ai**: Data curation; writing—original draft. **Hongyan Li**: Data curation; writing—review and editing. **Yong Hao**: Supervision; writing—review and editing. **Xiaoying Yao**: Project administration; supervision; writing—review and editing. **Yangtai Guan**: Funding acquisition; project administration; supervision.

## CONFLICT OF INTEREST STATEMENT

The authors declare no conflict of interest.

## ETHICS STATEMENT

The study was registered with the Chinese Clinical Trial Registry (CHICTR.org.cn) on 2 March 2016 (registration No. ChiCTR‐INR‐16008037), and the revised trial protocol (Protocol version 1.2.1) was released on 16 March 2020. The study protocol has been approved by the Ethics Committees of Renji Hospital (2016‐071K). All participants provided informed consent for enrollment in the study.

## Data Availability

The data that support the findings of this study are available from the corresponding author upon reasonable request.
